# Assessment of the impact an educational intervention on post-traumatic stress disorder and social cognitive theory constructs in women with sexual assault experience: a study protocol for a clinical trial

**DOI:** 10.1186/s12978-024-01866-2

**Published:** 2024-10-08

**Authors:** Nasrin Vafaeinejad, Zahra Boroumandfar, Ashraf Kazemi, Hamid Nasiri Dehsorkhi, Sosan Sohrabi

**Affiliations:** 1 Faculty of Nursing and Midwifery, Isfahan University of Medical Sciences, Isfahan, Iran; 2 Midwifery and Reproductive Health Department, Reproductive Sciences and Sexual Health Research Center, Isfahan University of Medical Sciences, Isfahan, Iran; 3 Midwifery and Reproductive Health Department, Isfahan University of Medical Sciences, Isfahan, Iran; 4 Clinical Psychology, Isfahan University of Medical Sciences, Isfahan, Iran; 5 Legal Medicine Research Center, Isfahan Forensic Medicine Organization, Isfahan, Iran

**Keywords:** Social-cognitive theory, Sexual assault, Post-traumatic stress disorder, Education

## Abstract

**Background:**

The high psychological burden of rape, together with complications such as pregnancy and chronic conditions, is associated with an increase in mental disorders. Social cognitive theory (SCT) is an important health theory that views behavior as dynamic and influenced by environmental, behavioral and individual factors. The aim of the present research is to determine the effect of an educational intervention on post-traumatic stress disorder and social cognitive theory constructs in women who have experienced sexual assault.

**Methods/design:**

This study is randomized, double-blind clinical trial research that will be conducted on 40 women with experience of sexual. In this research, by the intervention program based on social cognitive theory include" awareness, self-efficacy, outcome expectations and environment. Written consent will be obtained from the participants to participate in the research. Participants in the intervention group will be taught about health, hygiene, psychology and stress reduction methods in group or individual sessions according to their preference in 6 sessions of 60 min each, one session per week. A post-test will be conducted for both groups.

**Discussion:**

This study provides comprehensive data on the effect of providing an educational intervention using the social cognition Theory. Social cognitive theory focuses on how patterns of behavior are learned and how they function in interaction between the individual and the environment. It seems that the use of this theory in designing the content of educational interventions can be useful and practical.

*Trial registration*: The trial is prospectively recorded at the IRCT registry (Trial ID: IRCT20230926059526N1. Date recorded: 18/10/2023.

## Introduction

Sexual assault against women is an important public health and human rights issue with heterogeneous prevalence around the world [1]. Social inequalities and the cultural structures of societies play a role in the creation, perpetuation and survival of this issue [2]. Although the formation provided by virtual campaigns and the sharing of victims' experiences has increased public awareness of the problems and complications of sexual assault in recent years [3], in countries such as Iran, victims experience a very high psychological and social burden. This is because these individuals are not only the victims of an act of violence, but also bear the social pressure caused by the taboo of sexual assault taboo in society. In many cases, these victims do not report the assault to legal and health authorities and are therefore deprived of sexual health preventive measures. There are no clear and official statistics and information on sexual assault and physical abuse in Iran. More than 80% of cases of sexual harassment go unreported and the real statistics are higher than the published statistics [4]. According to the National Sexual Violence Resource Center, one in five American women will experience sexual assault at some point in her life [5]. Khoramin et al. indicated in a study that the prevalence of post-traumatic stress disorder in rape victims referred to forensic medicine in Kohgiluyeh and Boyar Ahmad provinces was almost 92% [6]. The results of the research on post-traumatic stress disorder among those referred to forensic centers in Tehran indicated that the rate of post-traumatic stress disorder and the rate of depression were respectively, 61.6% and 47.7% among the survivors [7]. The high psychological burden of rape, together with complications such as pregnancy and chronic conditions such as chronic pelvic pain, is associated with an increase in mental disorders. In cases where women have experienced more severe assaults, they show more symptoms of psychological distress [8]. Therefore, sexual assault as a crisis-causing event is associated with post-traumatic stress disorder, a complication that hinders the follow-up of diagnostic and therapeutic measures in victims [9]. In addition to the disturbed mental state of the victims, other factors also fuel this problem. Research has shown that there is a low level of awareness of sexually transmitted infections among the rape survivors attending the forensic medical centers. Based on the results of this study, 59% of the participants had no knowledge of the nature, mode of transmission and prevention of sexually transmitted infections, and 24% had low knowledge [10]. These findings show that rape victims in Iran are mostly unaware of the dangers of rape and may not take health-related measures despite the high post-traumatic stress they experience due to the social pressure caused by the taboo of being a rape victim. The interactions of individuals with the legal, medical and healthcare systems and the search for help after an accident have an impact on their mental well-being. Seeking help after the sexual assault is described by victims as a second rape, and many of these victims do not receive comprehensive care [11, 12]. Given the high economic and social burden of sexual assault on individuals and society, it is recommended that guidelines for accessing essential medical, legal, social support and reporting services be provided and established [1, 2]. To prevent post-traumatic stress disorder in 10 women who had recently been sexually assaulted, Fao et al. in a study designed a comparative program between an intervention group that received a cognitive-behavioral program and a control group that underwent an assessment of trauma-related psychological pathology. The results indicated that two months after the rape, women in the intervention group had fewer symptoms of post-traumatic stress disorder, and after five and a half months, the intervention group had less depression than the control group [13]. Other studies have used various methods such as client-centered therapy [14], mental imagery, and reprocessing of nightmares and suicidal thoughts [15] to reduce symptoms of psychological distress and depression in women who have experienced sexual assault. The individual, situational and cultural factors that contribute to the blaming of rape survivors should be considered in the design of studies and interventions [16]. Social Cognitive Theory [SCT] is an important health theory that views behavior as dynamic and influenced by environmental, behavioral and individual factors. Given that rape is a social problem, educational interventions that focus on the interpersonal level, such as those based on social cognitive theory, may be effective in resolving conflicts caused by the psychological pressures of rape on victims. It also appears that the constructs of this theory can be used to change a one's behavior, awareness and ability [17–19]. Investigating the effect of long-term exposure therapy on social support and the symptom severity in patients with post-traumatic stress disorder, Aghmiuni et al. found that the mentioned exposure therapy can alleviate post-traumatic stress disorder [20]. Studies in Western societies have shown that social support is an effective factor in reducing symptoms of post-traumatic stress disorder caused by sexual assault [21–23]. Cultural factors, economic conditions, family situation, existing legal/punitive systems [24] and the different societal views of women who have experienced sexual assault in Western and traditional societies are among the factors that make the impact of social support different. This is because the disclosure of this incident may expose women who have experienced sexual assault in traditional societies to particular social challenges and social stigma, thus questioning the impact of social support on PTSD in traditional societies [25]. Various measures have been taken to reduce the symptoms and severity of post-traumatic stress disorder. However, there is a continuing need for training programs and more research into post-traumatic stress disorder caused by the experience of sexual assault. The reduction of violence against women and its complications and the achievement of gender equality are among the world's development goals. Awareness-raising and training of women is one of the key aspects in achieving this goal. The aim of the present research is to determine the effect of an educational intervention on post-traumatic stress disorder and social cognitive theory constructs in women who have experienced sexual assault. In case of appropriate impact, the study can be used as an intervention to reduce individual and social complications, and economic costs. It can also promote health behavior change and the management of post-traumatic stress disorder, and encourage healthcare-seeking, thereby improving the quality of life of women who have been raped. This study determines and compares the background characteristics and the mean scores of post-traumatic stress, awareness, health-related attitudes and behaviors, perceived social support, and self-efficacy in and between two intervention and control groups before and two months after the intervention.

## Methods/design

The overall aim of the study is to designing and determining the effect of a training program based on social cognitive theory on post-traumatic stress disorder in women who experienced sexual assault. More specifically we will:

Question

1. What are the individual characteristics of the research units in the two intervention and control groups?

Aims.

1. Determining and comparing the average score of awareness, attitude and performance of health-related behavior in post-traumatic stress disorder of women who experienced sexual assault between the test and control groups before and two months after the intervention.
2. Determining and comparing the average score of perceived social support in post-traumatic stress disorder in women who experienced sexual assault between the test and control groups before and two months after the intervention.
3. Determining and comparing the average score of self-efficacy in post-traumatic stress disorder in women who experienced sexual assault between the test and control groups before and two months after the intervention.
4. Determining and comparing the average score of post-traumatic stress in women who experienced sexual assault between two test and control groups before and two months after the intervention.

### Study design

This study is randomized, double-blind clinical trial research that will be conducted on 40 women with experience of sexual assault referred to Iran-Isfahan Welfare and Forensic Medicine Centers. In the first phase of the educational intervention, the program, content and questionnaire will be designed and compiled, and in the second phase, sampling will be performed in the form of a census and, then, training sessions will be held. For the purpose of randomization, table of random numbers will be used for two intervention and control groups with a ratio of 1:1. The design of this clinical trial follows the requirements of the CONSORT statement.

#### Participants, recruitment, and study area

The statistical population of the study includes all women who, as a result of experiencing sexual assault, have referred to the Social Emergency and Forensic Medicine Centre of Isfahan City for support and through legal centers related to sexual assault, and whose referrals have been recorded. The study sample consists of women with experience of sexual assault who are referred to social counseling centers and forensic medicine in the city of Isfahan and who meet the conditions to participate in the study. The participants will be contacted using their recorded phone number.

#### Inclusion criteria

1. Women aged 18–45 years.
2. Women with at least one experience of sexual assault with an interval of at least one month from the incident [if there are symptoms of the disorder, no maximum], and obtained a score of 33 and above in PCL-5 questionnaire with the ability to diagnose based on the DSM-5 diagnostic system and the PTSD was confirmed in them by a clinical psychologist.
3. No current drug use.
4. No current major brain disease.
5. No mental retardation.
6. No major psychiatric disorder.
7. No treatment process.
8. No psychiatric history.
9. No psychiatric record [psychotic disorders: psychosis, bipolar disorder, schizophrenia, bipolar I disorder with psychotic symptoms, dementia], and no pregnancy at study entry.

#### Exclusion criteria

1. Missing more than one training session.
2. Experiencing another crisis during the study [mourning the death of a loved one, pregnancy, bankruptcy of the loved ones] were the exclusion criteria.

#### Randomization and data management

Owing to the limited number of samples in this research, census sampling method will be used by telephone invitation to all participants who meet the inclusion criteria. The names and phone numbers of the female survivors of sexual assault who were referred to the Social Emergency and Forensic Medicine of Isfahan City and who have files will be extracted from the archive and in coordination with the admission officer of the centers, and they will be invited to participate in the study. We will use the table of random numbers to randomly assign the participants to the two groups of intervention and control based on a one-to-one ratio. The participants’ recruitment will begin in April 2024, and will end in January 2025.

### Description of the intervention

#### Intervention group

In this research, by the intervention program based on social cognitive theory we mean the design and development of educational content based on this theory, using the opinion of experts in the field of clinical psychology, reading articles and psychology books, considering the nature of theoretical structures, and using different methods for the intervention group in order to focus on the individual's behavior in the areas of behavioral, environmental and individual factors. Social cognitive learning theory is concerned with the learning of patterns of behavior and the interaction between the individual and the environment. Thus, not only does the individual's response to the environment play a central role, but intellectual processes, i.e. cognitive operations resulting from human interaction with the environment and intrapersonal factors also play a fundamental role. The selected constructs in this research are awareness, self-efficacy, outcome expectations and environment, which will be used in the design of the intervention program focusing on health behavior change. In this study, environmental constructs and self-efficacy will be examined under the heading of perceived social support and perceived self-efficacy using standard questionnaires.

#### First phase: design and development of the program and content

In order to design the study and the content of the educational intervention based on the selected constructs of social cognitive theory, a search will be conducted in various sources of national and international articles using keywords such as sexual assault, education, social cognitive theory, sexual violence and post-traumatic stress disorder. In addition, psychology books and theories of midwifery, women and health will be examined.

An educational intervention will be carried out for the intervention group taking into account the nature of the theoretical constructs and using different methods to focus on individual behavior in the areas of behavioral, environmental and individual factors. The selected constructs in this study include awareness, self-efficacy, outcome expectations and environment, which will be used in the design of the intervention program focusing on health behavior change. Environmental constructs and self-efficacy will be examined under the titles of perceived social support and perceived self-efficacy using standard questionnaires. The intervention is designed for 40 participants, and for validation the opinions of expert professors and the research team will be used, and after their approval the intervention will be started. Education is delivered by the student and the research clinical psychologist using approved content during sessions.

#### Second phase: implementation of the intervention and training sessions

The instructor is a Master of Forensic Midwifery student and all training is delivered under the supervision and approval of the research team and the research team's clinical psychologist, and the content is approved by the research team. Participants in the intervention group will be taught about health, hygiene, psychology and stress reduction methods in group or individual sessions according to their preference in 6 sessions of 60 min each, one session per week. After obtaining the ethics permission from Isfahan University of Medical Sciences and the IRCT code, a letter of introduction is obtained to be submitted to Isfahan Welfare and Forensic Medicine Organizations. Written permission will be obtained from the officials to enter and access the medical files, and the researcher will be present in the research environment and introduced to the officials. The objectives of the research and the method of doing the study will be explained. An educational intervention is designed and developed, taking into account the constructs of social cognitive theory and focusing on health-related behavior. Sampling will take the form of a census of the participants who meet the eligibility criteria after accessing the file in the above-mentioned centers, and they will be invited to participate in the study by telephone. The participants are randomly assigned to the intervention and control groups using a random number table. Before participating in the research, they will be given a description of the research objectives, the intervention method and sufficient assurances of confidentiality, and they will be able to choose whether to participate in group or individual sessions to receive the educational intervention. Written consent will be obtained from the participants to participate in the research, and pre-test will be performed. Participants allocated to the intervention group will be invited to participate in four groups of 5 participants according to their age and education, or if they do not wish to be in a group, they will be invited to attend individual sessions. Then, an educational intervention based on social cognitive theory will be implemented in the Welfare and Forensic Medicine Organizations during 6 sessions of 45–60 min once a week and for 6 weeks by a trained instructor. The qualification of this instructor is confirmed by the consulting professor, Dr. Nasiri, [PhD in clinical psychology] during a three-session program with health-related educational content. In order to provide the educational intervention [attached], the student will conduct the intervention based on social cognitive theory constructs in sessions under the supervision and approval of the team psychologist, and two sessions will be carried out in the presence of the professor. The educational content has been prepared on the basis of theoretical constructs and using the scientific techniques available in psychological articles and books, and taking into account the conditions required for the training, and will be analyzed and approved by the supervisor and advisor. In order to make the intervention in the direction of social support and environment, according to the specific social conditions, educational brochures will be given to the individuals to have the influence of the environment, so that they can request social support from the supportive people or give the brochure to their supporters and receive the education indirectly. It should be noted that in parallel with the intervention group, in order to double-blind the control group, a one-hour session for the control group will be provided with the routine training of the above-mentioned centers, without focusing on specific theoretical structures and continuous follow-up of sexually transmitted diseases. During the study, they will use the services and routine care provided by the centers, they will not be deprived of these services, and the researcher will not know about the groups. The time required for the research process [proposal preparation and approval, sampling and intervention, final report and article preparation, article editing and submission] is 14 months. Brochures, booklets and CDs with the same content as the sessions are used as teaching methods to make the sessions more accessible. For two months after the end of the intervention, the participants will be followed up, if necessary, through a telephone line, virtual group and SMS, and their questions will be answered. A post-test will be conducted for both groups to check the effect of the educational intervention two months after the last intervention session in the intervention group. This study is conducted to measure the effect of the independent variable (intervention training) on the dependent variables health-related awareness, health-related attitude and behavior, self-efficacy, perceived social support and PTSD severity score of women with experience of sexual assault. It also aims to reduce the symptoms of stress disorder, increase awareness, and improve health-related attitude and behavior, self-efficacy and perceived social support. The variables of background characteristics, self-efficacy, perceived social support, post-traumatic stress disorder, awareness and health-related attitudes and behaviors will be measured using a self-report questionnaire before and two months after the intervention in two groups. The measurement tools used in this research include a questionnaire on demographic characteristics, a standard questionnaire on self-efficacy, a standard questionnaire on perceived social support, PCL-5 post-traumatic stress disorder questionnaire, and a researcher-made questionnaire on health-related awareness, attitude and behavior. The questionnaire will be filled as self-report, and if a participant is illiterate, it is read out to them. It is hoped that by using the constructs of social cognitive theory in the content of training sessions based on scientific documents, it will be possible to meet the needs of these participants and prevent further injury and problems.

### Control group

**Fig. 1:**
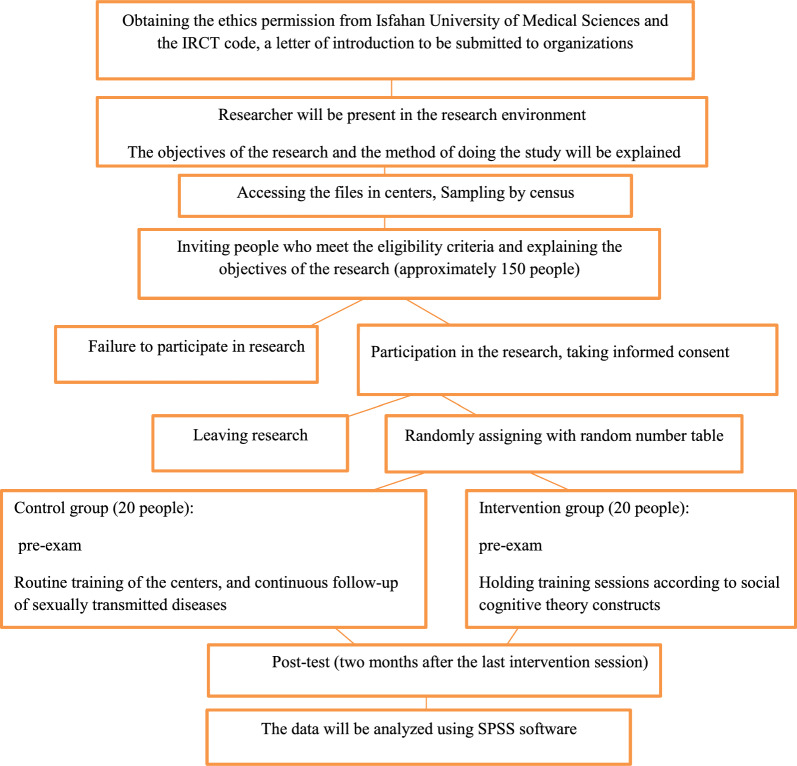
Flow diagram of the participants through the trial

The control group will continue to receive routine care, plus ongoing training on how to follow up sexually transmitted infections. The routine care that is done in our country is that, if a woman is sexually assaulted, she should first go to the police headquarters. Then the police introduce her to one of the court's branches that deals with rape issues. The judge introduces her to the Forensic Medicine, where the investigation of the raped woman is conducted, and the evidence and documents of the court case are presented, and according to the administrative procedures, they try to reach the rapist. At the same time, if the woman is raped, she will be introduced to a counselor who is present in the court so that she can be consulted and taken care of. Any type of health care (HIV test, prophylactic medication for STI, etc.) is done by raped women in private centers or comprehensive health service centers. Figure 1 details the flow of participants from enrollment until the last follow-up contact for control and intervention subjects.

### Sample size

According to the mean values and standard deviation before and after the educational intervention in previous studies [26] and asking the opinions of statistics experts and considering α = 0.05 and β = 0.2, the standard deviations of 7.95 and 8.06 in the intervention and control groups, and a minimum difference of 7.5 points between the two groups in terms of post-traumatic stress disorder, the sample size is calculated to be 18 participants in each group. However, considering a drop-out rate of 10%, the sample size is set at 20 participants in each group.

#### Data collection tools and methods

After the woman is randomized to the control or intervention group and has signed the written consent, having been previously informed of the study aims, the researcher will let her know about the follow-up she will need to complete during the study. The measurement tools used in this research include a questionnaire on demographic characteristics, GSE-10 self-efficacy questionnaire, a questionnaire on perceived social support, PCL-5 post-traumatic stress disorder questionnaire, and a researcher-made questionnaire on health-related awareness, attitude and behavior.

The Demographic Characteristics Questionnaire covers demographic information such as age, economic status, education level, occupation, marital status, number of children if married, spouse's education and occupation, residential status, birth order, parents' education and health insurance status. The number of supporters, number of rapes, previous experience of rape, relationship with the rapist, number of rapists and beatings during the event can be recorded using the researcher-made checklist. For example, "what is your birth order in your family?".

To examine perceived social support, the Multidimensional Perceived Social Support Scale is used, which measures social support from three sources of family, society and friends, using 12 items on a 7-point Likert scale ranging from “strongly disagree” (score 1) to “strongly agree” (score 7). The minimum and maximum scores for a person's perceived social support on the whole scale are 12 and 84, respectively, with the higher the score, the greater the social support. The validity and reliability of the tool has been checked and confirmed by Besha rat [27, 28]. A sample item is "There is a special person in my life who helps me when I need help", and is measured on a 7-point Likert scale.

Post-traumatic stress disorder in the present study refers to a score confirmed by the PLC-5 questionnaire and the opinion of a clinical psychologist. The PCL-5 is a self-report psychometric measure of PTSD symptoms according to the DSM-5, developed by Weds et al. [29]. The PCL-5 is a 20-item questionnaire, with a 5-point Likert scale (0–4) including 0( not at all), 1 (a little bit), 2 (moderately), 3 (quite a lot) and 4 (extremely) and the total score is calculated with a range of 0–80. Higher scores indicate a greater degree of severity of PTSD in the individual. The validity and reliability of the Persian version was assessed and confirmed by Vermaghani et al. in Iran. The optimal cut-off points for identifying possible positive cases of PTSD in the Iranian population are considered to be 33 [30]. The Cronbach's alpha of the English version of this instrument is 0.95 and the French version is 0.94. The validity and reliability of this instrument in Iran was assessed by Vermaghani et al., and the Cronbach's alpha coefficient for the Persian version was reported to be 0.92 [31, 32]. The questionnaire has been validated by the research team. An example of a checklist item is "extremely negative emotions such as fear, anger, guilt and shame", which is determined by a score of 0 to 4.

Self-efficacy in this research is the score that participants will receive on the General Self-Efficacy Scale (GSE-10). This questionnaire was developed and revised by Schwarzer and Jerusalem with the aim of assessing general self-efficacy [33, 34]. This questionnaire was implemented in Iran by Rajabi [35] on 587 students of Shahid Chamran University of Ahvaz and Marvdasht University. This scale has 10 items on a Likert scale from 1 to 4, with the lowest and highest scores being 10 and 40, respectively. The scale is used to predict adjustment after life transitions or as an index of how life is going at each stage of development, for clinical work and for behavior modification. The Cronbach's alpha coefficient of the GSE-10 scale in Iran is 0.84, which is higher than the standard in different countries, suggesting that it has a high reliability. A sample item of the scale is "If I try hard enough, I can always solve difficult problems", which is measured on a Likert scale of 1–4 ranging from "completely true" to "not true at all".

To measure the health-related awareness, attitudes and functioning, a researcher-made questionnaire was designed and developed using articles and psychology books. This questionnaire contains 21 items and includes 7 questions on awareness, 7 questions on attitudes and 7 questions on functioning in relation to health. To assess validity and reliability of the questionnaire, the final review will be conducted by 11 professors from Isfahan School of Nursing and Midwifery, Department of Health Psychology, Social and Forensic Medicine, and an epidemiologist. For example, a health-related awareness question is "There are drugs that can prevent the occurrence of sexually transmitted infections" that is scored 1 for a correct answer and 0 for an incorrect answer and I don't know. A health-related item of attitudes is "I am afraid to talk about sexual assault with my relatives", which is measured on a 5-point Likert scale. An example of a health-related behavior item is "To check for complications after rape, I do a genital examination", which is measured using a 4-point Likert scale. Data collection will be performed using a questionnaire on demographic characteristics, GSE-10 self-efficacy questionnaire, a questionnaire on perceived social support, PCL-5 post-traumatic stress disorder questionnaire, and a researcher-made questionnaire on health-related awareness, attitude and behavior. If the research samples did not have a correct understanding of the meaning of the word at the time of completing the questionnaire, it would be explained to them by the questioner.

### Unfavorable events

Unfavorable events are presently not predicted, due to the study and intervention’s nature.

### Analysis

All statistical trials and confidence intervals will be supposed with a type I error established in alpha = 0.05, using the IBM SPSS V26 statistics package. The data in this research are of a quantitative and qualitative nature, and descriptive and inferential statistical methods are used for analysis. In order to achieve the research question, determine and compare the demographic characteristics (field) between the two test and control groups, from descriptive statistics tests, independent t-test for quantitative variables such as age and number of family members, for nominal qualitative variables such as occupation. Chi-square test was used and Mann–Whitney test was used for the qualitative variable, level of education, type of residence, child's birth rate. In order to achieve the objectives of the research, the analysis of variance with repeated measures will be used to investigate the main and combined effects, and then the Bonferroni post hoc test will be used to answer the objectives (comparison within each group) and objectives (comparison between two groups).

## Discussion

Sexual assault has many effects on mental and physical health and is linked to various personal, social and environmental issues. Besides physical complications, sexual assault causes psychological and social damage. Since the complications caused by sexual assault include the damage to a girl's virginity, it can lead to psychosocial complications in traditional societies and add to its psychological complications. These complications, as gender-related complications, can in turn affect a person's sexual identity. The social consequences of sexual assault are a serious problem for the female survivors in traditional societies, as they are often reluctant to disclose the incident and therefore do not receive social support from their families. Accordingly, this poses a serious challenge to their access to reproductive and mental health services. The possibility of pregnancy is another serious problem for these women, and the lack of access to legal abortion can lead these women to the process of illegal abortion and its complications. The lack of awareness of health rights and legal and civil protection and their negative attitude towards seeking health services due to the fear of disclosing rape, together with the reduction in receiving and seeking social support among these women and their families, can explain the possibility of the effectiveness of the program based on social cognitive theory. It is expected that prevention strategies, resources and early treatment, as well as strategies for rehabilitation, education and awareness of reproductive and sexual health, will be provided in order to prevent the health-related disruption rape can cause and its negative consequences [36, 37]. Because rape causes physical, mental and emotional damage, many countries have taken various measures to change the law and increase support for sexual assault survivors, including the establishment of rape crisis clinics. However, in our country, in addition to the weaknesses in the laws relating to sexual assault and how to prove it, no measures have been taken regarding support and compensation for emotional and psychological damage, and in many cases, victims receive no support and are unable to return to their normal lives [38]. Affected by gender stereotypes and the culture of patriarchal societies, women are considered to be the weak and second sex, and their position has been weakened. Efforts to eliminate stereotypes and raise awareness can help to purge societies of sexual violence [39]. Lack of attention to the psychological needs and treatment of individuals who have experienced sexual assault can lead to them becoming secondary victims and [40]. The essence of humanity is the ability to exercise control over life and to have personal, environmental and social agency to control one's personal destiny and social life. Social cognitive theory is an important theory that considers all three dimensions of person, environment and society [41]. Based on this theory, people can help to improve their life and create social changes [42]. Social cognitive theory focuses on how patterns of behavior are learned and how they function in interaction between the individual and the environment. Thus, not only does the individual's response to the environment play a central role, but intellectual processes, i.e., cognitive operations resulting from human interaction with the environment, and intrapersonal factors also play a fundamental role [43]. Social cognitive theory examines various structures of awareness, outcome expectations, situational awareness, environment, self-efficacy, self-efficacy in overcoming obstacles, self-control or goal setting, and emotional coping [44, 45]. It seems that the use of this theory in designing the content of educational interventions can be useful and practical.

## Conclusion

This study provides important information about the clinical effectiveness of educational interventions as part of PTSD treatment. It is expected that the present study will show that educational intervention is relatively inexpensive and can help support individuals.

### Trial status

This research was registered in Iran Clinical Trials Registration IRCT20230926059526N1 on 26 October 2023. To date, 40 participants have been randomly selected. It is expected that the recruitment of participants will be completed in March 2024.

## Data Availability

No datasets were generated or analysed during the current study.

## Abbreviations

PTSD: Post traumatic stress disorder
SCT: Social cognitive theory
